# Supplementary material for: S.M.A.R.T. F.U.S: Surrogate Model of Attenuation and Refraction in Transcranial Focused Ultrasound
Source: PLoS One. 2022 Oct 27;17(10):e0264101. doi: 10.1371/journal.pone.0264101 (PMC9612531; doi:10.1371/journal.pone.0264101)
Supplement: S1 Fig — This figure shows the axial pressure (Pa) derived from SMART_FUS and O’Neil water simulations when using the same input parameters (in this case, a focal depth of 50mm and diameter of 60mm). Note that the x axis depicts the distance from the back of the transducer’s concavity and thus the focus is more than 50mm from this point. The noise between 0 and 15mm on the x axis in the SMART_FUS derived pressure map is also related to the fact that these values come from within the transducer concavity itself. (DOCX) [file pone.0264101.s001.docx]

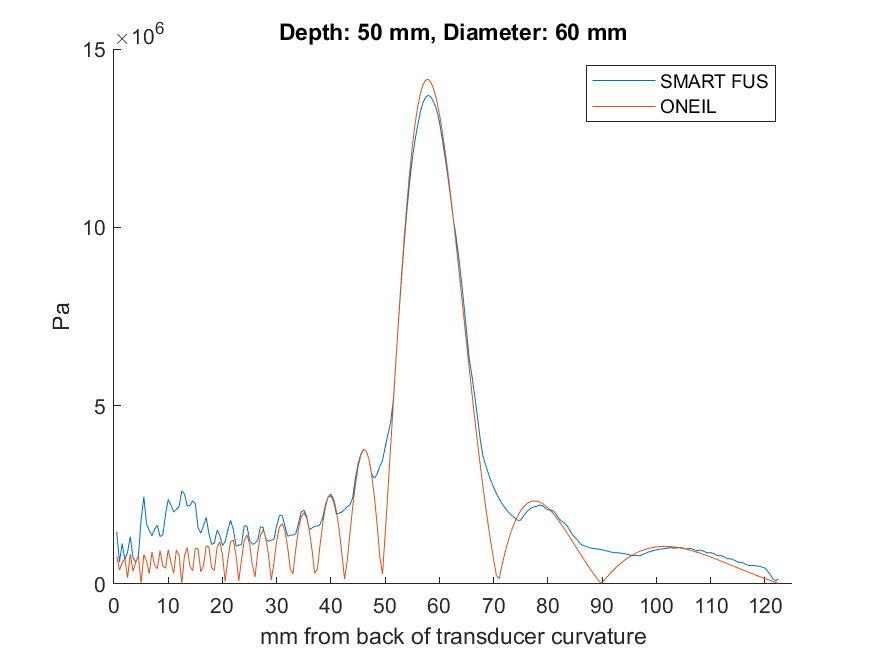


**Figure A1: O’Neil Comparison Example.** This figure shows the axial pressure (Pa) derived from SMART_FUS and O’Neil water simulations when using the same input parameters (in this case, a focal depth of 50mm and diameter of 60mm). Note that the x axis depicts the distance from the back of the transducer’s concavity and thus the focus is more than 50mm from this point. The noise between 0 and 15mm on the x axis in the SMART_FUS derived pressure map is also related to the fact that these values come from within the transducer concavity itself.
