# Supplementary material for: S.M.A.R.T. F.U.S: Surrogate Model of Attenuation and Refraction in Transcranial Focused Ultrasound
Source: PLoS One. 2022 Oct 27;17(10):e0264101. doi: 10.1371/journal.pone.0264101 (PMC9612531; doi:10.1371/journal.pone.0264101)
Supplement: S1 Table — CFL’s of 0.1, 0.2, and 0.3 were used while PPW was varied between 3 and 6 by doubling dimensions of the simulation space for half of the simulations. All other parameters were held constant at 1 MHz Fundamental frequency, 12mm Bone thickness, 90 degrees trajectory (flat against bone), 80mm transducer depth and 80mm transducer width. The attenuation and refraction across this smaller parameter space is provided. (DOCX) [file pone.0264101.s002.docx]

| **Sim. Parameters** | **Refraction** | **Attenuation** |
| --- | --- | --- |
| 6ppw CFL 0.1 | 17mm | 71.79% |
| 6ppw CFL 0.2 | 16.75mm | 67.68% |
| 6ppw CFL 0.3 | 16.75mm | 63.04% |
| 3ppw CFL 0.1 | 17.5mm | 69.85% |
| 3ppw CFL 0.2 | 17.5mm | 67.83% |
| 3ppw CFL 0.3 | 18mm | 68.67% |

**Table A1: Convergence Test.** CFL’s of 0.1, 0.2, and 0.3 were used while PPW was varied between 3 and 6 by doubling dimensions of the simulation space for half of the simulations. All other parameters were held constant at 1 MHz Fundamental frequency, 12mm Bone thickness, 90 degrees trajectory (flat against bone), 80mm transducer depth and 80mm transducer width. The attenuation and refraction across this smaller parameter space is provided.
