# Supplementary material for: S.M.A.R.T. F.U.S: Surrogate Model of Attenuation and Refraction in Transcranial Focused Ultrasound
Source: PLoS One. 2022 Oct 27;17(10):e0264101. doi: 10.1371/journal.pone.0264101 (PMC9612531; doi:10.1371/journal.pone.0264101)
Supplement: Attachment — Submitted filename: Reviewer3.docx [file pone.0264101.s003.docx]

Reviewer #3:

This is a short manuscript that introduces two matlab functions to aid in transcranial focused ultrasound. The functions are idealized functions of a single bone type of a flat configuration. As such a simplified model of bone, it is not clear what the application for these matlab functions would be. A better understanding of the intended use of the functions is needed here. A clear use case would be helpful.

We appreciate the concern surrounding the highly simplified bone layer. This was an important consideration during the planning of this work. As staircasing has been found to introduce considerable error, we found that avoiding it would be preferable to, e.g., emulating a slightly curved structure more closely resembling a human skull.

As stated in the manuscript, running simulations which mimic or represent the real experimental setup—including the position of ultrasound transducer and the subject’s skull shape(including thickness)— is preferable once these values are known. However, we believe the value of this work is the estimation of skull attenuation/refraction prior to knowing these value, for instance, in the planning phase of an experiment where one may be considering trajectories through the human skull that the results of SMART_FUS may advise against considering the degree of attenuation/refraction expected.

Indeed, given great individual differences in skull shape which may be present directly under the transducer when positioned for any given stimulation session with any given individual, it seems impossible to better estimate that shape prior to subject recruitment. Instead, we opt here to simply represent bone at a given width, which we believe can be more readily estimated for a given planned trajectory (e.g., into the central thalamus through the temporal bone) using the known ranges of human skull thickness and the known position of brain regions of interest. Again, a flat bone structure avoids the staircasing necessary for more complex skull models, which was found to introduce the most error among possible error-inducing factors for similar simulations using K-wave (Robertson et al., 2017; cited in main text).

In addition, some details of the functions are not clear. What is “trajectory?” Is this angle with respect to the bone surface?

Trajectory is explained on line 130 in the main text but has also been explained on line 95 in the edited text.

What was the rationale and references for the choices of bone acoustic properties?

These are explained in the last paragraph of the methods section with references to the sources of the parameters used.
